# Development and characterization of microsatellite markers for endangered species *Stipa pennata* (Poaceae) and their usefulness in intraspecific delimitation

**DOI:** 10.1007/s11033-018-4192-x

**Published:** 2018-05-21

**Authors:** Ewelina Klichowska, Monika Ślipiko, Marcin Nobis, Monika Szczecińska

**Affiliations:** 1 Institute of Botany, Faculty of Biology, Jagiellonian University, Gronostajowa 3, 30-387 Kraków, Poland; 2 Department of Botany and Nature Protection, Faculty of Biology and Biotechnology, University of Warmia and Mazury, Plac Łódzki 1, 10-728 Olsztyn, Poland

**Keywords:** *Stipa*, Genetic diversity, SSR, Intraspecific delimitation, Illumina HiSeq

## Abstract

*Stipa pennata* (Poaceae), has become a rare and endangered species in Central Europe due habitat loss and fragmentation. This species is characterized by high morphological variability, which has resulted in the description of numerous intraspecific taxa. The aim of present work is to develop microsatellite markers useful in population genetics studies as well as in intraspecific taxonomy of *S. pennata* s.l. We developed ten microsatellite markers using Illumina high-throughput. Polymorphism at each marker was evaluated using 4–15 individuals from four morphotypes of *S. pennata* s.l. Seven markers showed polymorphism while three were monomorphic. The number of alleles per locus ranged from 7 to 12, and the observed and expected heterozygosity varied from 0.000 to 1.000 and 0.000 to 0.8670, respectively. Our results confirm that three of four studied morphotypes are genetically distinct. The microsatellite markers developed here will be useful for evaluating levels of genetic diversity and differentiation, to study gene flow, population dynamics and in future conservation studies as well as for intraspecific delimitation of morphologically similar taxa within *S. pennata* s.l.

## Introduction

*Stipa* L. is one of the largest genera in the family Poaceae comprising over 150 species distributed in open grasslands and steppes, with the highest species diversity in the warm temperate regions of the Old World [1]. One of the most widely distributed species of the genus, is *Stipa pennata* L. [syn. *S. joannis* Čelak.], a perennial grass, occurring mostly in dry grasslands and steppes of Europe and Asia [2]. Over the last few decades, changes in land use in Europe, connected mostly with abandonment of grasslands and agricultural intensification has resulted in xerothermic habitats fragmentation and loss [3]. In the last few decades most of European countries noted a significant decrease in both, number of individuals and number of populations of *S. pennata*. Currently the species is protected and red-listed in many European countries [e.g. 2, 4–7].

Because of the high morphological variability, many lower rank taxa have been described within *S. pennata* s.l., e.g.: *S. pennata* var. *okensis* (P.A. Smirnov) Tzvelev, *S. joannis* f. *subpuberula* Podpěra & Suza, *S. joannis* var. *puberula* Podpěra & Suza, *S. disjuncta* Klokov, *S. graniticola* Klokov, *S. pennata* subsp. *ceynowae* Klichowska & M. Nobis [8, 9].

Due to high rates of mutation, high level of polymorphism as well as codominant inheritance, microsatellites are useful in reconstructing the relatively recent genetic processes occurring in populations [10]. They are the most popular markers used to determine the genetic diversity and differentiation of populations of rare and endangered species [11–15]. Moreover, some studies have postulated their suitability for phylogenetic reconstruction and taxonomic delimitation [16–18]. To the best of our knowledge, microsatellite markers were specifically developed only for two species of *Stipa* – *S. breviflora* Griseb. [19]. and *S. purpurea* Griseb. [20].

The aim of this study was to develop for the first time microsatellite markers for *S. pennata*, using high-throughput Illumina sequencing. Our second aim was evaluating the suitability of newly developed markers for population genetic studies as well as intraspecific delimitation. To this end, we tested our microsatellite markers in four morphotypes (taxa) from *S. pennata* s.l.: morphotype 1—typical *S. pennata* with short prickles at the adaxial surface of vegetative leaves, glabrous cauline leaf sheets and short ligules of leaves of vegetative shoots, morphotype 2—with long hairs at the adaxial surface of vegetative leaves, morphotype 3—with cauline life sheets shortly pubescent, and morphotype 4—with long ligules of the vegetative leaves.

## Materials and methods

Plant materials were collected in Poland from four distant populations of *Stipa pennata* s.l., one per each morphotype (taxon). For each population we sampled from 4 to 15 individuals (small numbers of individuals results from a small population size). At least one voucher per population is deposited at the Herbarium of the Institute of Botany, Jagiellonian University (KRA), Kraków, Poland. Total genomic DNA was extracted from dry leaf tissue using the Genomic Mini AX Plant Spin (A&A Biotechnology, Gdynia, Poland). DNA quantity was estimated using Qubit fluorometer (Invitrogen, Carlsbad, NM, USA).

We constructed a genomic library using a TruSeq Nano DNA Library kit (350 bp insert size; Illumina, San Diego, CA, USA). The library was sequenced by Macrogen, South Korea (https://dna.macrogen.com/), using 100 bp paired-end reads on an Illumina HiSeq 2000 platform (Illumina, San Diego, CA, USA). The obtained pair-end 100 bp reads were cleaned by removing low quality (Q below 5), short (< than 50 bp) and unpaired reads. Plastid reads were removed by mapping onto to previously published *Stipa* plastomes [21] using Geneiuos 7.01 (Biomatters, New Zealand) mapper with medium/low sensitivity settings. The remained reads were assembled de novo using Velvet [22]. Analysis of 4653 contings from 500 to 108,820 bp using MSATCOMMANDER software identified 322 SSR motifs in 320 contigs. Among identified SSR motifs we designed 57 primers. The ten microsatellite loci showed a clear, single peak for each allele. These ten loci were subsequently used to screen 43 individuals representing different morphotypes of *S. pennata* s.l. PCR reactions were performed in 20 µl of reaction mixture, containing 40 ng genomic DNA, 1x PCR buffer, 1 µM of each primer, 1 µl BSA, 200 µM dNTP, and 1U RUN DNA Polymerase (A&A Biotechnology, Gdynia, Poland). All candidate primer pairs were tested under the following thermal conditions: (1) initial denaturation, 4 min at 94 °C, (2) denaturation, 30 s at 94 °C, (3) annealing, 30 s at 57–63 °C, (4) elongation, 1 min at 72 °C, and final elongation, 7 min at 72 °C. Stages 2–4 were repeated 35 times. PCR products were separated on a Qiaxcel capillary electrophoresis system, using the Qiaxcel High Resolution Kit with the alignment marker 15–500 bp and the DNA size marker pUC18/HaeIII for microsatellites (Qiagen, Hilden, Germany). Standard OM700 settings were used as the electrophoresis program [23]. Automatic sizing of the amplified fragments was performed using a PC running BioCalculator software according to the manufacturer’s instructions (Qiagen, Hilden, Germany).

Genetic diversity estimates were calculated using GenAIEx 6.41 [24]. Deviations from the Hardy–Weinberg equilibrium (HWE) and linkage disequilibrium between loci were tested using FSTAT 2.9.3 [25]. Significance levels were adjusted using Bonferroni correction for multiple testing. The sequences of the SSR fragments were deposited in the GenBank (Table 1). Principal coordinate analysis (PCoA) based on Fst genetic distances was performed using GenAIEx 6.41 [24].

**Table 1:** Characteristics of 10 microsatellite loci developed for *Stipa pennata* s.l.

Locus	Primer sequences (5′-3′)	Repeat motif	Allele size range	A	Ta (^o^C)	GenBank accession no.
*SP10*	F:CGCCTTTGTTGTTTATGAGCAG R:AGCTAGTGTCCCACGTGTC	(TA)_7_	165–185	9	62	MG978348
*SP12*	F:TAGATACGCCGGCTCGTT R:GTGATGGCAAGTACGGCAG	(GCCC)_4_	401–420	12	60	MG978349
*SP41*	F:GGAAAGATGCGACAACCCG R:AACTTGAGCAGCCTCTTGG	(GAA)_4_	412	1	58	MG978355
*SP17*	F:ACTGTTGAAACCACGATCCG R:GCGGAACATTTGCCTTTGG	(TAA)_4_	326–350	11	58	MG978351
*SP43*	F:GGCAGAACAAATGGAGCCC R:GCAAACGCATCGAAACCTC	(AAT)_4_	323	1	57	MG978356
*SP23*	F:CTTAGCGCCTGGCCAAATC R:CCTTTCCTGAAGCTAAACCGAC	(TA)_6_	297–309	10	63	MG978352
*SP28*	F:AGGCTCAGTGTCCGCAGAAG R:AGGCATAGCCAAATGCCAC	(TC)_6_	237–243	7	60	MG978353
*SP30*	F:AAAGCGGACGGCATTGTTC R:AGAAAGCAAGCTTACGGTGC	(TA)_7_	210	1	57	MG978354
*SP08*	F:CCGGAAATACAATATCCTACCGC R:GTCCGGAGGTCTCTCAAGG	(CAA)_3_	288–297	7	57	MG968959
*SP15*	F:AGCGTAAAGCTCTCGAGTATG R:CGAAGGGAGTCGCAAATTCAC	(TTA)_4_	413–430	7	59	MG978350

*A* number of alleles sampled; *Ta* annealing temperature

## Results and discussion

In the studied populations, seven loci showed polymorphism with 7 to 12 alleles per locus, while three loci were monomorphic. Significant numbers of those loci (6/10) contained tri- or hexa-nucleotide repeats (Table 1). All ten markers were successfully amplificated for all studied morphotypes (Table 2). The highest average number of alleles per locus (4.4) were detected for the morphotype 1 whereas the lowest average number of alleles (2.2) was found in population of morphotype 4. The observed heterozygosity and expected heterozygosity of each population (for polymorphic loci) ranged from 0.000 to 1.000 and 0.000 to 0.8670, respectively (Table 2). Significant deviations (p < 0.05) from Hardy–Weinberg equilibrium (HWE) due to homozygote excess were detected for locus *SP08* in the morphotype 2 and for *SP10, SP17* and *SP23* in morphotype 4, which suggests the presence of null alleles. Despite the small number of studied individuals, we obtained similarly high genetic diversity, compared to other species of the genus *Stipa* [19, 20], which confirms usefulness of these markers for population studies.

**Table 2:** Genetic variation of 10 microsatellite loci of *Stipa pennata* s.l.

Locus	Morphotype 1				Morphotype 2				Morphotype 3				Morphotype 4			
	Przyłubie (n = 15)^a^				Toruń–Barbarka (n = 10)^a^				Pamięcin (n = 14)^a^				Folusz (n = 4)^a^			
	A	Ae	He	Ho	A	Ae	He	Ho	A	Ae	He	Ho	A	Ae	He	Ho
*SP10*	7	5.696	0.067	0.824	5	2.941	0.700	0.660	4	3.161	0.000	0.684	2	2.000	1.000	0.500*
*SP12*	3	2.273	0.000	0.560	6	5.000	0.113	0.800	5	4.083	0.000	0.755	1	1.000	0.000	0.000
*SP41*	1	1.000	0.000	0.000	1	1.000	0.000	0.000	1	1.000	0.000	0.000	1	1.000	0.000	0.000
*SP17*	6	3.846	0.234	0.733	5	2.817	0.290	0.645	5	3.664	0.214	0.727	3	1.852	0.200	0.460*
*SP43*	1	1.000	0.000	0.000	1	1.000	0.000	0.000	1	1.000	0.000	0.000	1	1.000	0.000	0.000
*SP23*	9	7.500	0.133	0.867	5	3.846	0.200	0.700	9	4.612	0.214	0.783	3	1.515	0.200	0.340*
*SP28*	5	3.600	0.136	0.722	6	3.125	0.200	0.680	4	2.142	0.143	0.533	3	2.632	0.200	0.620
*SP30*	1	1.000	0.000	0.000	1	1.000	0.000	0.000	1	1.000	0.000	0.000	1	1.000	0.000	0.000
*SP08*	2	2.000	1.000	0.500	4	2.410	0.876	0.760*	2	2.000	0.768	0.500	6	4.545	1.000	0.780
*SP15*	9	6.429	0.000	0.844	5	3.125	0.000	0.680	3	2.579	0.110	0.612	1	1.000	0.000	0.000

*A* number of alleles; *Ae* effective number of alleles; *He* expected heterozygosity; *Ho* observed heterozygosity; *n* number of individuals sampled for each population

*Significant deviation from Hardy–Weinberg equilibrium (p < 0.05)

^a^The locations of the populations are as follows: Poland, Przyłubie, 53.028499N/18.369725E; Poland, Toruń, Barbarka, 53.039342N/18.548170E; Poland, Pamięcin, 52.465616N/14.666387E; Poland, Folusz near Szubin, 52.978472N/17.704709E

**Fig. 1:**
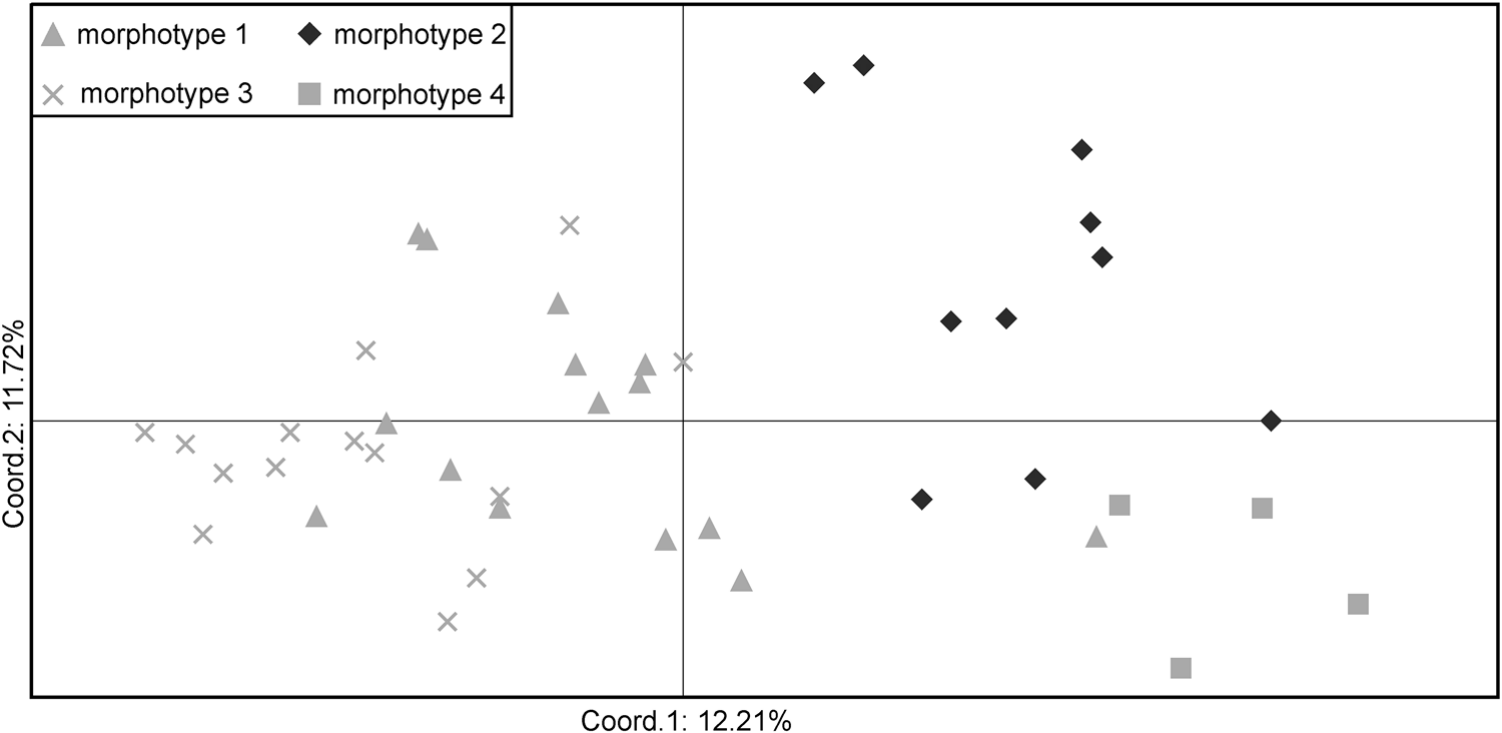
Principal coordinate analysis (PCoA) based on Fst genetic distances for populations of *Stipa pennata* s.l.

Principal coordinate analysis (PCoA) based on seven loci (Fig. 1) demonstrated, that it is possible to distinguish three out of the four studied morphotypes (taxa) from *S. pennata* s.l. The first axis, which explained 12.21% of the total variance, separated populations of morphotype 2 and morphotype 4 from other morphotypes. The second axis (explained 11.72% of variance) separated morphotype 2 from morphotype 4. These results confirm that newly developed markers can be used to a certain degree for intraspecific delimitation. It seems to be particularly useful in the case of the genus *Stipa*, in which numerous taxa of lower rank have been described [8].

## Conclusions

Markers presented here can be used for evaluating genetic diversity within and between populations, gene flow between populations of *S. pennata* as well as population dynamics. Developed primers could be used for conservation genetic studies of this rare and endangered species. These markers can be also useful for clarifying the genetic boundaries between morphologically difficult to distinguish, intraspecific taxa (morphotypes).

## References

[1] Nobis M (2014). Taxonomic revision of the Central Asian *Stipa tianschanica* complex (Poaceae) with particular reference to the epidermal micromorphology of the lemma. Folia Geobot.

[2] Ceynowa-Giełdon M, Nobis M, Rutkowski L, Kaźmierczakowa R, Zarzycki K, Mirek Z (2014). *Stipa pennata* L.—Ostnica piórkowata. Polska czerwona księga roślin: paprotniki i rośliny kwiatowe.

[3] Janišová M, Bartha S, Kiehl K, Dengler J (2011). Advances in the conservation of dry grasslands: introduction to contributions from the seventh European Dry Grassland Meeting. Plant Biosyst.

[4] Korneck D, Schnittler M, Vollmer I (1996). Rote Liste der Farn- und Blütenpflanzen (Pteridophyta et Spermatophyta) Deutschlands. Schr-reihe f Vegetationskunde.

[5] Iliashenko VY, Iliashenko EI (2000). Krasnaya kniga Rossii: pravovye akty [Red Data Book of Russia: legislative acts].

[6] Procházka F (2001). Černý a červený seznam cévnatých rostlin České republiky (stav v roce 2000). Príroda Praha.

[7] Didukh Y (2009). Red book of Ukraine. Plants. Part 1.

[8] Gonzalo R, Aedo C, García MA (2013). Taxonomic revision of the Eurasian *Stipa* subsections *Stipa* and *Tirsae* (Poaceae). Syst Bot.

[9] Klichowska E, Nobis M (2016). *Stipa pennata* subsp. ceynowae (Poaceae, Pooideae), a new taxon from Central Europe. PhytoKeys.

[10] Freeland JR, Kirk H, Petersen S (2012). Molecular ecology.

[11] Provan J, Wilson PJ (2003). Effect of habitat fragmentation on levels and patterns of genetic diversity in natural populations of the peat moss *Polytrichum commune*. Proc R Soc B.

[12] Williams BL, Brawn JD, Paige KN (2003). Landscape scale genetic effects of habitat fragmentation on a high gene flow species: *Speyeria idalia* (Nymphalidae). Mol Ecol.

[13] Ortego J, Aguirre MP, Noguerales V, Cordero PJ (2015). Consequences of extensive habitat fragmentation in landscape-level patterns of genetic diversity and structure in the Mediterranean esparto grasshopper. Evol Appl.

[14] Cleary KA, Waits LP, Hohenlohe PA (2016). Development and characterization of fourteen novel microsatellite markers for the chestnut short-tailed fruit bat (*Carollia castanea*), and cross-amplification to related species. PeerJ.

[15] Szczecińska M, Sramko G, Wołosz K, Sawicki J (2016). Genetic diversity and population structure of the rare and endangered plant species *Pulsatilla patens* (L.) Mill in East Central Europe. PLoS ONE.

[16] Ochieng JW, Steane DA, Ladiges PY, Baverstock PR, Henry RJ, Shepherd M (2007). Microsatellites retain phylogenetic signals across genera in eucalypts (Myrtaceae). Genet Mol Biol.

[17] Turini FG, Steinert C, Heubl G, Bringmann G, Lombe BK, Mudogo V, Meimberg H (2014). Microsatellites facilitate species delimitation in Congolese Ancistrocladus (Ancistrocladaceae), a genus with pharmacologically potent naphthylisoquinoline alkaloids. Taxon.

[18] Miz RB, Tacuatiá LO, Cidade FW, de Souza AP, Bered F, Eggers L, de Souza-Chies TT (2016). Isolation and characterization of microsatellite loci in Sisyrinchium (Iridaceae) and cross amplification in other genera. Genet Mol Res.

[19] Ren J, Su Z-Z, Dang Z-H, Ding Y, Wang P-X, Niu J-M (2017). Development and characterization of EST-SSR markers in *Stipa breviflora* (Poaceae). Appl Plant Sci.

[20] Liu W, Liao H, Zhou Y, Zhao Y, Song Z (2011). Microsatellite primers in *Stipa purpurea* (Poaceae), a dominant species of the steppe on the Qinghai-Tibetan Plateau. Am J Bot.

[21] Krawczyk K, Nobis M, Myszczyński K, Klichowska E, Sawicki J (2018). Plastid super-barcodes as a tool for species discrimination in feather grasses (Poaceae: *Stipa*). Sci Rep.

[22] Zerbino DR, Birney E (2008). Velvet: algorithms for de novo short read assembly using de Bruijn graphs. Genome Res.

[23] Wang X, Rinehart TA, Wadl PA, Spiers JM, Hadziabdic D, Windham MT, Trigiano RN (2009). A new electrophoresis technique to separate microsatellite alleles. Afr J Biotechnol.

[24] Peakall R, Smouse PE (2006). GenAlEx 6: genetic analysis in Excel. Population genetic software for teaching and research. Mol Ecol Notes.

[25] Goudet J (2001) FSTAT, A program to estimate and test gene diversities and fixation indices, version 2.9.3. University of Lausanne, Lausanne. https://www2.unil.ch/popgen/softwares/fstat.htm. Accessed 8 Feb 2018

